# CJD mimics and chameleons

**DOI:** 10.1136/practneurol-2016-001571

**Published:** 2017-02-02

**Authors:** Simon Mead, Peter Rudge

**Affiliations:** NHS National Prion Clinic, National Hospital for Neurology and Neurosurgery, University College London Hospitals NHS Foundation Trust, London, UK

**Keywords:** rapidly progressive dementia

## Abstract

Rapidly progressive dementia mimicking Creutzfeldt–Jakob disease (CJD) is a relatively rare presentation but a rewarding one to become familiar with, as the potential diagnoses range from the universally fatal to the completely reversible. Patients require urgent decisions about assessment and investigation and have quickly evolving needs for treatments and support, through symptom management and end-of-life care in most cases. We have based this pragmatic review on the experiences of a specialist prion referral centre in the UK, which, unsurprisingly, is strongly biased towards seeing patients with CJD. Cases eventually proven not to have prion disease might be described as ‘CJD-mimics’; being referred from UK neurologists, these are the most challenging cases. CJD in its classical presentation is very rarely mimicked; however, it is highly heterogeneous, and atypical forms can mimic virtually all common neurodegenerative syndromes. Warning features of a mimic include generalised seizures, hyponatraemia, fever, a facial movement disorder, a normal neurological examination and a modestly rapid presentation. Contrast-enhancing lesions or MRI signal hyperintensity outside the striatum, thalamus or cortex and a cerebrospinal fluid pleocytosis are key investigation pointers to a CJD mimic.

## Introduction

Rapidly progressive dementia (RPD) is a clinical syndrome that is not well defined and little studied. There are some excellent and comprehensive reviews,^1–3^ including by Murray,^4^ in this journal. The archetypal diagnosis is Creutzfeldt–Jakob disease (CJD), and most experiences have been reported from specialist prion disease referral centres and from a small number of specialist cognitive clinics. Some have used a definition of a diagnosis of dementia within 1 year of symptom onset, although this is problematic, as initial symptoms are often non-specific and merge with highly prevalent complaints in the population; therefore, the exact time of onset is hard to pinpoint. Nevertheless, the concept of RPD is sensible, as it differentiates a set of syndromes and diagnoses that are, to a significant degree, distinct from those seen in the differential diagnosis of common neurodegenerative disorders. CJD is associated with progression from normal function to *death* in under a year in approximately 90% of patients.^5^ Those reporting the history of patient with CJD witness a cognitive and neurological disorder leading to obvious progressive changes in everyday functions over periods of weeks or a month’s duration, rather than noticing change over 6 months to a year, which would be more typical of a common dementia.

There are no population studies of RPD, and the experience in prion disease referral centres is likely to be substantially different from the reality in primary and secondary care centres. Referral patterns vary considerably between countries, based on historical factors and the services provided by a specialist clinic. The proportion of RPD patients eventually diagnosed as prion disease varies in reports from 20% to the large majority (>80% in our case).^1 6^ This variation relates to the considerable amount of filtering done by local physicians before patients are referred.

Undoubtedly, many patients with rapid changes in their function and dementia have a delirium, recognised by acute or subacute deficits in attention; concentration and orientation; a cognitive disorder and an explanatory toxic, metabolic or infective physiological problem (Diagnostic and Statistical Manual of Mental Disorders, Fifth edition). A key feature here is the identification through history taking of a pre-existing and more insidiously progressive cognitive disorder. Similarly, multiple strokes, viral encephalitis and brain tumours might meet our working definition of RPD but are readily identified at initial assessments. Here, we focus more on particularly difficult cases that have made it through the filters of local physicians and were therefore challenging to differentiate from CJD.

### What are the phenotypes of CJD?

Prion diseases are highly heterogeneous disorders with distinct causes, clinical features and durations.^7^ Most commonly (~85% of the annual incidence), the cause is unknown, termed ‘sporadic’ CJD. Acquired causes are sometimes immediately obvious, such as treatment with cadaveric pituitary-derived human growth hormone (before 1985), or using cadaver-derived dura mater to repair defects during neurosurgery (before 1992). Variant CJD, caused by the human transmission of the cattle prion disease bovine spongiform encephalopathy, involved much more widespread exposure of the UK and other populations, although specific at-risk groups, such as those patients who received a blood transfusion derived from a donor who later developed variant CJD, are identifiable. These individuals may have been notified of their increased risk status. The recognition of genetic causes may be prompted by evidence of a familial disorder; however, family history is often negative in definite cases, because for example, several causal genetic mutations typically manifest in old age and are partially penetrant. The range of clinical phenotypes of inherited prion disease extends far beyond RPD, and investigation results are much less specific than for CJD; consequently, our practice for all undiagnosed cases that we see is to screen the only gene that contains mutations that cause inherited prion disease, the prion protein gene (*PRNP*).

In advanced stages, all prion diseases look very similar: the patients have akinetic mutism. They lie still in a hospital bed, often have eyes open but not tracking a face moving across the visual field, have occasional spontaneous or startle-related myoclonus, are incontinent and silent or make only incomprehensible noises. If swallowing is retained and depending on other aspects of supportive care, patients can survive in this state for several weeks, even years, with tube feeding. In sporadic CJD, patients deteriorate to this state as rapidly as in a few weeks in the fastest cases or over a year in 10%.^8^ In the vast majority of cases, patients are admitted for urgent inpatient assessment following the first interview in the hospital, and investigations leading to diagnosis are organised in a single hospital stay. Many patients are discharged from this first hospital stay into 24-hour care or a hospice.

**Figure 1:**
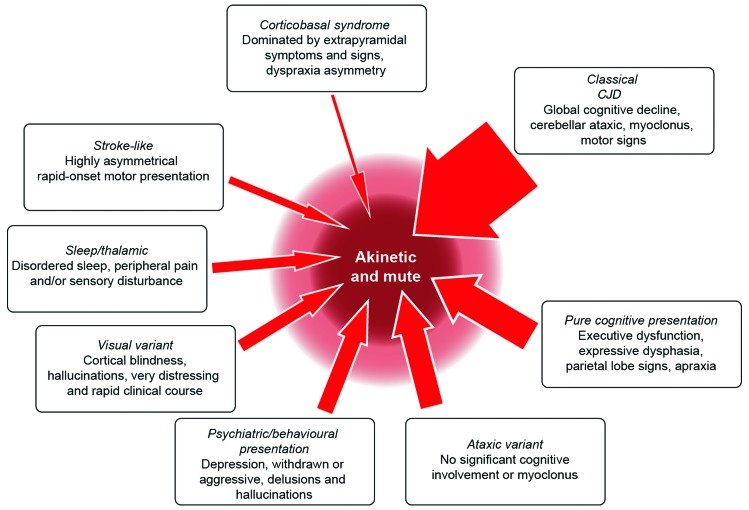
Creutzfeldt-Jakob disease (CJD) clinical features and progression. The boxes describe clinical variants of CJD. The width of each arrow relates to the proportion of cases with the presentation. Patients with CJD become more similar over time, and almost all enter a phase of ‘akinetic mutism’ before death.

The first symptoms of sporadic CJD are usually non-specific: headache, malaise, cough, dizziness and change in personality, mood or memory. The first more CJD-specific symptoms are diverse and lead to distinct initial differentials (figure 1). In half of the cases, the more typical picture of CJD is present on exploring the history further or reveals itself during examination or shortly after an initial assessment. In its *classical presentation* (figure 1), the clinical syndrome of CJD comprises an RPD (widespread cognitive domains: memory, dysexecutive, behaviour disturbance, abnormalities of calculation and spelling and dysphasia^9^) associated with cerebellar ataxia, myoclonus, pyramidal and extrapyramidal motor signs and often evidence of a disorder of higher visual functions. Aside from rapid forms of common neurodegenerative diseases, the main differentials are limbic, corticosteroid-responsive or infective encephalitis, paraneoplastic conditions, Wernicke’s encephalopathy, neurosarcoidosis, hypothyroidism, non-convulsive status epilepticus, hypoxic encephalopathy, a toxic/metabolic syndrome or a functional disorder.

Atypical presentations cause more difficulty, including pure *cognitive presentations* (~15%) that might be mistaken for rapid forms of Alzheimer’s disease, frontotemporal dementia or dementia with Lewy bodies; *ataxic presentations* (~10%) are often associated with acquired CJD and might be mistaken for disorders of the cerebellum and its brainstem connections, including vascular, neoplastic, paraneoplastic or inflammatory conditions. The *visual presentation* or Heidenhain variant (~5%) is particularly distressing for patients with hemianopia or scotoma, misperceptions, hallucinations, distortions and palinopsia. Progression tends to be rapid, even relative to other presentations of CJD. The condition often presents to ophthalmologists or optometrists as an ocular disorder, for example, suspected cataract.^10^ *Psychiatric presentations* (~5%) also occur; thus, in variant CJD, depression and personality change are common early in the evolution of the disease, while paranoia, visual hallucinations and aggression occasionally occur during the initial phase of the disease in sporadic CJD, suggesting a primary psychiatric diagnosis of psychosis or depression.^11^ Very rare presentations include those that mimic a *stroke* (~2%) or *corticobasal syndrome* (~2%). The *thalamic presentation* (~2%) includes sleep disturbance and distal pain and may include abnormalities of the autonomic nervous system: palpitations, temperature dysregulation, hypertension or postural hypotension. This presentation is well known as fatal familial insomnia and is typically associated with the inherited prion disease caused by the D178N missense mutation in *PRNP*, usually linked to a methionine allele at polymorphic codon 129; however, there have been cases without this or any gene mutation.

While we list the obvious conditions that a neurologist would consider after an initial assessment, it is usually clear that the patient warrants urgent and comprehensive investigation. At an initial assessment, the rapidity of progression may not be evident, either because there is no witness account available or because comorbidities or prodromal symptoms obscure the underlying disease. Some interventions are therefore indicated immediately while waiting for initial test results. Suspect cases of viral encephalitis should be treated with intravenous acyclovir. Cases associated with weight loss, alcohol excess or other evidence of malnutrition should be treated with intravenous vitamins. In all patients, we recommend a blood screen, including routine haematology, biochemistry, thyroid function, vitamin B_12_, serology for neurosyphilis, paraneoplastic antibodies and serology for limbic encephalitis; MR scan of brain (dementia protocol), including axial diffusion-weighted imaging and fluid-attenuated inversion recovery (FLAIR); electroencephalograph (EEG) and cerebrospinal fluid (CSF) examination for basic constituents, 14-3-3 protein, S100b, abeta and tau proteins, and the real-time quaking-induced conversion assay (RT-QUIC) (table 1).

**Table 1:** Investigations/work-up

**Screen in all CJD mimic patients**	**Second-line test**
**Basic blood tests:** routine haematology, biochemistry, calcium, magnesium, vitamin B_12_, HIV serology, thyroid function, antinuclear antibody, antineutrophil cytoplasmic antibody, inflammatory markers	Serum ammonia, drug levels (as appropriate)
**Serology:** Voltage-gated K-channel antibodies, NMDA-R antibodies, TPHA (neurosyphilis)	Serology for specific infections (eg, Lyme), paraneoplastic antibodies
**Imaging:** MR scan of brain, dementia protocol, including diffusion-weighted axial images and post-gadolinium imaging for lymphoma/inflammatory CNS conditions	Angiography (vasculitis, dural arteriovenous fistula, intravascular lymphoma)
**EEG:** routine recording	Electromyography and nerve conduction studies
**CSF:** for cells, biochemistry, oligoclonal bands, 14-3-3 protein, S100b protein, RT-QUIC assay, amyloid beta protein (1–42), total tau	CSF serology for NMDA-R encephalitis, measles serology, PCR for JC virus and for viruses associated with other encephalitides
**PRNP:** gene analysis	Dementia gene panel diagnostics
**Urine:** test for infection	Brain biopsy

CJD, Creutzfeldt–Jakob disease; CNS, central nervous system; CSF, cerebrospinal fluid; EEG, electroencephalogram; JC, John Cunnigham, NMDA-R, *N*-methyl-D-aspartate; *PRNP*, prion protein gene; RT-QUIC, real-time quaking-induced conversion assay.

### Investigation features of CJD

#### Imaging

**Figure 2:**
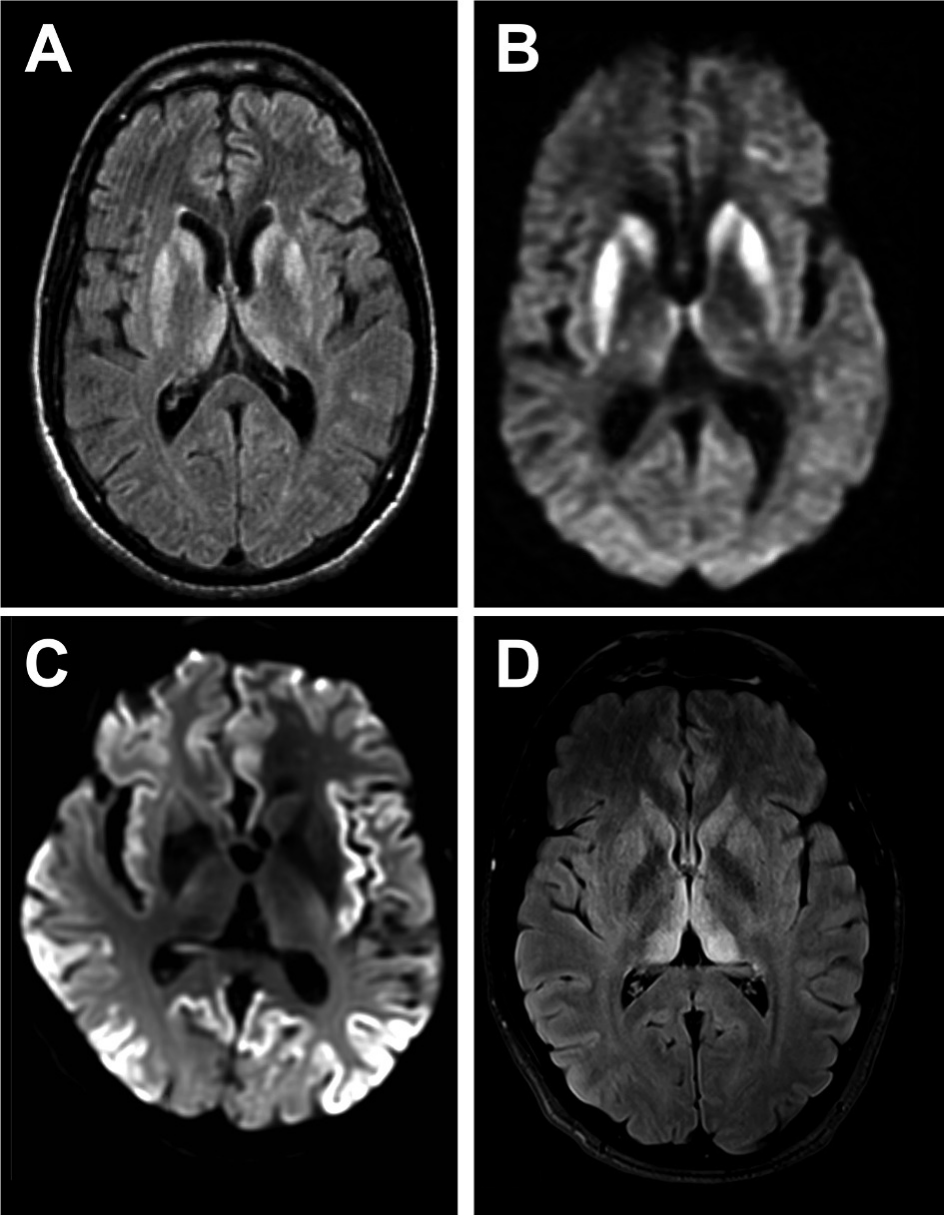
Typical MRI features of Creutzfeldt-Jakob disease (CJD). (A and B) Sporadic CJD showing typical basal ganglia signal return on fluid-attenuated inversion recovery (FLAIR) (A), which is more obvious on diffusion-weighted sequences (B). (C) Diffusion-weighted imaging sequence showing striking cortical ribboning with normal basal ganglia in sporadic CJD. (D) Variant CJD showing pulvinar sign on the FLAIR sequence.

MR imaging of brain is an extremely useful investigation in CJD, because it is readily available at short notice and non-invasive.^12^ Classical abnormalities are high signal on diffusion-weighted imaging or FLAIR in the striatum, cerebral cortex and/or thalamus (figure 2). Changes at these three sites may occur in isolation or combination. Cortical signal change is often patchy and extensive but should involve more than one cortical region and areas that are not vulnerable to artefactual signal change (eg, the frontal pole) and should not enhance or show mass effect. Thalamic signal change may be diffuse or have emphasis in dorsal and medial aspects but should not be of greater signal intensity than in the striatum in sporadic CJD. Most units report a sensitivity of MRI>90% in the diagnosis of sporadic CJD. In grey matter, CJD is associated with small vacuoles in neuronal cell bodies, axons or dendrites, which are most probably the pathological substrate for imaging abnormalities. Often, cortical signal change correlates with clinical features, for example, contralateral in the frontal lobe in patients with hemiparesis or occipital cortex signal change in the visual variant of CJD. Post-gadolinium imaging can help identify blood–brain barrier breakdown in lymphoma and neuroinflammatory conditions, which is not seen in CJD.

#### Electroencephalogram

The EEG is less useful with the availability of very specific imaging and CSF tests. Periodic sharp wave complexes develop in half of the patients with sporadic CJD, particularly in the later stages. It is most useful when showing unexpected findings that would be unusual in CJD and provide suggestive evidence for alternative diagnoses. These include periodic lateralising epileptiform discharges in encephalitis, frontal intermittent delta activity in toxic–metabolic or structural abnormalities (if asymmetrical), status epilepticus or runs of 2–3 s of polyphasic slow/sharp waves (subacute sclerosing panencephalitis). The extreme delta brush sign may occur in autoimmune encephalitis.

#### Cerebrospinal fluid

Pleocytosis in CJD can occur but is rare. For 20 years, the main CSF tests have been the detection of proteins released into the spinal fluid, as the disease process damages glia and neurones; these include 14-3-3, S100b and neurone-specific enolase.^13^ Over 80% of patients with CJD have abnormalities of these proteins, but importantly, they are not specific to the pathogenesis of CJD; rather, they reflect rapid tissue damage, and therefore, may also be abnormal in many of the mimic conditions. New CSF tests, such as the real-time quaking-induced conversion assay (RT-QUIC), are entering routine clinical use and initial evidence suggests these are much more specific.^14^ The RT-QUIC protocol involves disruption of prion protein aggregates in a test sample by rounds of shaking, followed by incubation in an excess of recombinant prion protein and induction of recombinant prion protein misfolding, detected by an increase thioflavin T fluorescence through the protocol (hence real time). CSF angiotensin-converting enzyme can help diagnose neurosarcoidosis.

#### Prion protein gene sequencing

Prion protein gene sequencing should, in our opinion, be routinely considered in all cases. The phenotypic spectrum of inherited prion disease is more diverse than CJD, and family history is often absent due to old-age presentations/partial penetrance. Most patients with CJD are homozygous at the common polymorphism at codon 129 of the gene. The fastest declining patients with CJD are typically methionine homozygous at this site, whereas the more slowly changing patients are usually methionine–valine heterozygous. While this is a powerful effect, ruling out inherited prion disease is the most important purpose for gene sequencing. Gene testing has implications for blood relatives, who must be involved in the decision to proceed.

#### Other tissues and biofluids

Tonsillar biopsy or a blood test (the Direct Detection Assay, from the National Prion Clinic) can be used in the diagnosis of variant CJD but has no use in the sporadic form. Prion protein amplification or capture diagnostic technologies may be used in the future, with material from olfactory brushings or using urine or blood, but for the time being, these tools are in research development for sporadic CJD.^15 16^ Brain biopsy is seldom used to make a diagnosis of CJD, because it is reserved for circumstances when a reversible diagnosis seems possible, in particular where a specific lesion is to be targeted. It is essential to take prion precautions before neurosurgery in suspect cases. For more detailed guidance, see https://www.gov.uk/government/publications/guidance-from-the-acdp-tse-risk-management-subgroup-formerly-tse-working-group.

### Mimics of CJD after initial investigation

Once initial investigations are done, patients who cause ongoing difficulty usually have abnormality of at least one of the key initial tests. Most frequently, this is a positive 14-3-3 protein in CSF.

#### Common neurodegenerative disorders

In over half of the mimics that the National Prion Clinic sees, the eventual diagnosis is another neurodegenerative disease. These conditions are much more prevalent than CJD and are also heterogeneous, with confusion only likely to occur with the very most rapidly progressing end of the spectrum of Alzheimer’s disease or dementia with Lewy bodies. Comorbidities, such as infection, leading to delirium, or drug changes are often a factor. Key features enabling recognition here are evidence from the witness account of pre-existing symptoms before the acute or subacute presentation. MR imaging evidence of focal or generalised atrophy or CSF abnormality of abeta or tau proteins are also instructive. The diagnosis becomes clear when patients stabilise or improve, which hardly ever happens in patients with CJD despite treatment of intercurrent infection. Vascular disease in the striatum, cortex or thalamus can also contribute to diagnostic confusion, because at first glance, this might suggest prion disease. Myoclonus, ataxia and motor signs can develop in common neurodegenerative diseases and can worsen with drug treatments, for example, tricyclics and myoclonus, antiepileptic drugs and ataxia, neuroleptics and dementia with Lewy bodies.

Although there are no specific disease-modifying therapies, accurate diagnosis of CJD can lead to meaningful management decisions about symptomatic therapies and choices for the patient and carers. All neurodegenerative disorders have Mendelian genetic causes, which (eg, in the case of *C9orf72* expansion) can be associated with rapid progression and motor signs that bring CJD into the differential. The availability of gene panel diagnostics will identify an increasing proportion of cases with a specific genetic diagnosis.^17^

We are particularly interested in potentially treatable mimics, which may be grouped into the following categories.

#### Immune-mediated encephalitis

Probably, the most important CJD mimics are the recently characterised antibody-mediated inflammatory disorders of the central nervous system.^18 19^ While some are paraneoplastic and fluorodeoxyglucose PET is useful in locating the underlying tumour, a substantial proportion is not associated with antibodies directed to a range of neuronal surface ion channels and membrane receptors. Many cases have dramatic or significant recovery following immune therapies.

**Figure 3:**
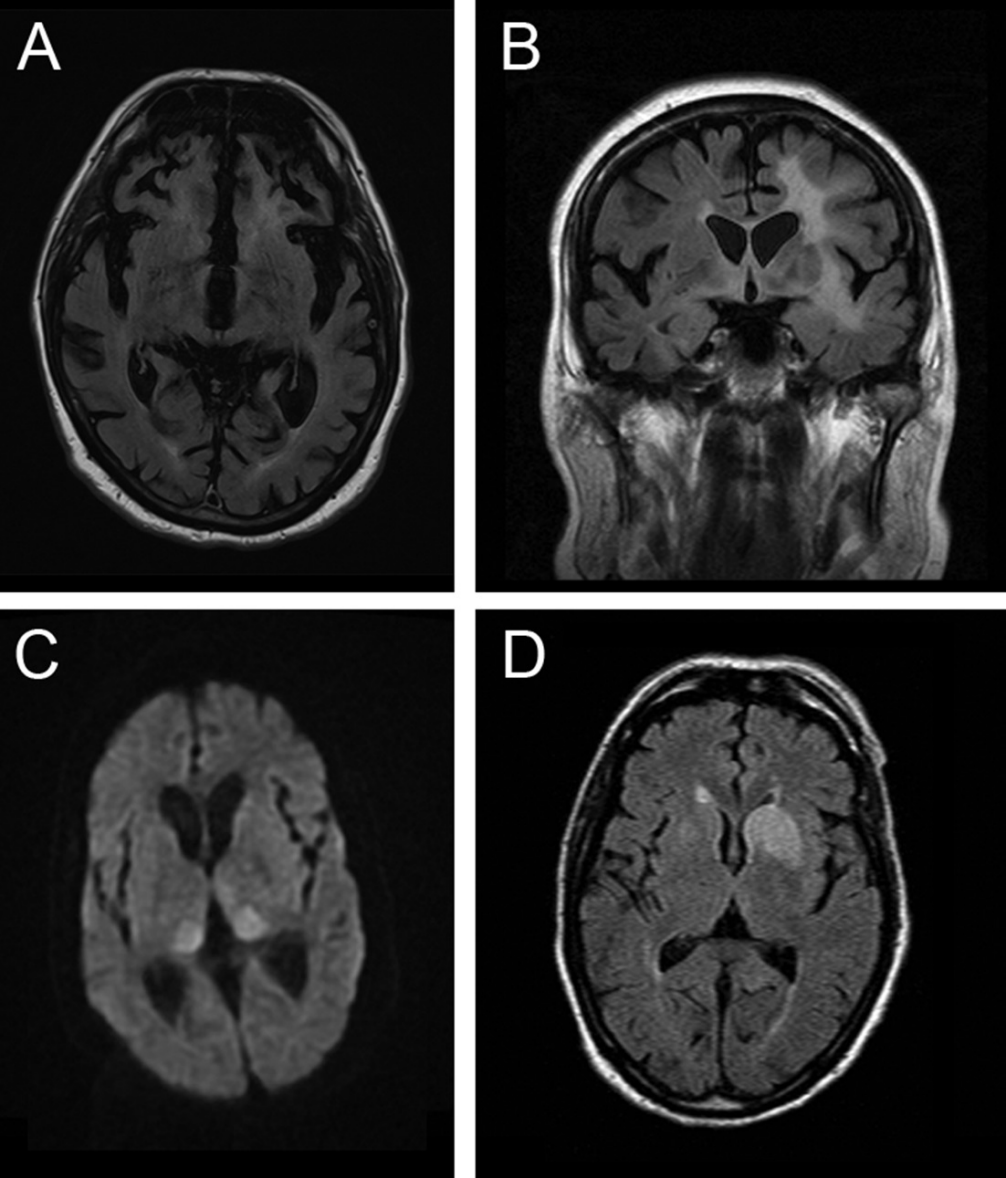
MR scan of brain in four patients presenting as mimics of prion disease. (A) *C9orf72* mutation showing severe generalised atrophy with no parenchymal abnormal signal. Severe atrophy does occur late in some cases of Creutzfeldt-Jakob disease (CJD), but usually diffusion-weighted sequences reveal restricted diffusion. (B) B-cell lymphoma confined at the time of presentation to the brain. Note the extensive white-matter signal change with the normal cortex. Occasionally, the leukoencephalopathic form of CJD has white-matter signal alteration, but this is associated with grey matter destruction. (C) *N*-methyl-D-aspartate antibody encephalitis with MR imaging showing the pulvinar sign. This sign develops in most cases of variant CJD and has rarely been described with other pathologies. (D) Voltage-gated potassium channel (Casp-1) antibody encephalitis showing unilateral basal ganglia high signal on diffusion-weighted imaging but not on the apparent diffusion coefficient map (scan not shown). Note that the swelling of the caudate is a feature that does not occur in CJD.

N*-methyl-*D*-aspartate receptor (NMDAR) antibody encephalitis* is the most commonly recognised type, initially linked to ovarian teratoma in young women, which remains a prompt to consider the diagnosis. Like in patients with CJD, the history often includes a non-specific prodromal stage, developing into behavioural and psychiatric features, and early generalised and focal seizures that would be very unusual in CJD. Movement disorders commonly develop in CJD, but the specific facial dyskinesia of NMDAR encephalitis would prioritise the disorder. Other flags for the disorder are a CSF pleocytosis, CSF oligoclonal bands, and MR imaging findings (Table 3). These can include T2 or FLAIR hyperintensity of the striatum, thalamus and cortex as might occur in CJD (figure 3) but more typically include abnormalities in other areas, particularly the mesial temporal lobe and hippocampus, and also the brainstem, cerebellum and white matter, often with enhancement or mass effect from inflammation. We recommend testing at least serum in all patients suspected to have CJD and CSF in patients with flags for the disorder.

*Voltage-gated potassium channel (VGKC) complex antibody encephalitis* is similarly a crucial mimic of CJD to exclude. Importantly, we now know that pathogenic antibodies are directed at individual members of the VGKC multiprotein complex, for example, LGI1, which may give more specific tests. While associated with a wide range of clinical syndromes, the most commonly confused picture with CJD is one that may include both peripheral manifestations—neuromyotonia, a twitching or stiffness related to spontaneous motor nerve excitability, and autonomic disturbances—and central manifestations, such as brief and frequent seizures affecting ipsilateral face and arm (termed ‘faciobrachial dystonic’), insomnia, memory impairment and behavioural disturbances or more florid manifestations of an encephalitis. Investigation prompts to the diagnosis include hyponatraemia and MR imaging abnormalities of the mesial temporal lobe and hippocampus, which can also involve the basal ganglia (figure 3).

Other protein targets in immune-mediated encephalitis are proliferating and include *glutamic acid decarboxylase,* which can be associated with limbic encephalitis or cerebellar ataxia; *AMPA receptor antibody encephalitis*, often associated with tumours, psychosis and limbic encephalitis; *GABA_A_ or GABA_B_R antibody limbic encephalitis; glycine receptor* antibodies, which may occur in progressive encephalopathy with generalised stiffness, autonomic abnormalities and myoclonus (progressive encephalomyelitis with rigidity and myoclonus); and *metabotropic glutamate receptor* antibodies, which have been reported in subacute cerebellar degeneration.

*Hashimoto’s encephalitis* is commonly referred to as a key differential of CJD, but we have never seen this at the Prion Clinic. Testing of thyroid function and antithyroperoxidase antibodies is simple to do, although many patients reported to have the disorder are euthyroid. The pathogenesis of this disorder and its distinction from other immune-mediated encephalitides is unclear.

#### Infections

*Progressive multifocal encephalopathy* is an opportunistic brain infection associated with the polyoma JC virus infection, almost exclusively in immunocompromised people. There is usually a history of immunosuppressive drug use, HIV, Hodgkin’s lymphoma or chronic lymphocytic leukaemia. The condition is hard to link to a specific symptom, although it is mistaken for CJD because of rapid progression of disability and its common involvement of visual pathways or cerebellar ataxia. As for encephalitis, the occurrence of generalised seizures in half of the cases would be unusual for CJD. Imaging features should be distinct from CJD, with a propensity for parieto-occipital demyelinating lesions at the junction of grey and white matter, less commonly seen in the corpus callosum, thalamus and basal ganglia. CSF is used to confirm diagnosis with PCR for JC virus, but a pleocytosis often occurs and would be another feature against a diagnosis of CJD.

*Subacute sclerosing panencephalitis* is a rare late complication of measles infection in early childhood. The clinical picture can look quite similar to CJD, with early lethargy and behavioural change followed by a ‘hung-up’ type of myoclonus, seizures, cognitive impairment, later motor signs and reduced level of consciousness. The diagnosis should be considered in children or young adults and may be prompted by EEG findings of bilaterally synchronous and repetitive sharp complexes lasting a few seconds. CSF antibody titres for measles are diagnostic.

*Other viral encephalitides* usually present acutely and are immediately recognised by general features such as fever and raised inflammatory markers in serum and CSF, along with more specific features of brain infection like seizures, meningism and coma. Occasionally, its more subacute presentations may cause some confusion with CJD.

A wide range of bacterial, fungal and parasitic infections that can cause an RPD should be on a reference checklist (table 2) but have not caused diagnostic confusion with CJD at our clinic.

**Table 2:** Differential diagnosis checklist

**Common disorders with rare presentation that mimic CJD**	**Rare but potentially treatable/reversible mimics**	**Rare and untreatable mimics of CJD**
Rapidly progressive forms of common neurodegenerative diseases	Limbic and other immune-mediated encephalitis	Unusual infections: subacute sclerosing panencephalitis
Delirium and pre-existing dementia	Metabolic and endocrine conditions (hyperammonaemia, electrolyte disturbance, hypoglycaemia/hyperglycaemia, uraemia)	Mitochondrial cytopathy
Viral encephalitis	Primary CNS vasculitis	Diffuse neoplastic disease (eg, carcinomatous meningitis, gliomatosis cerebri)
Hepatic failure and encephalopathy	Neurosarcoidosis	
Cerebrovascular disease (single or multiple strokes) or hypoxic encephalopathy	Primary CNS and intravascular lymphoma	
Wernicke’s encephalopathy and related manifestations of alcohol-related dementia (eg, extrapontine myelinolysis)	Unusual infections: progressive multifocal leukoencephalopathy, fungal encephalitis, Lyme disease, Whipple’s disease, neurosyphilis	
Thyroid dysfunction	Large dural arteriovenous fistula	
	­HIV-related dementia	
	­Subacute combined degeneration	
	­CNS manifestations of autoimmune disorders (eg, lupus, sarcoidosis)	
	­Heavy metal toxicity (eg, lithium, mercury)	
	­Toxicity related to medications (eg, lithium, valproate)	
	­Non-convulsive status epilepticus	
	­Psychiatric conditions: functional disorders, catatonia, depression	

CJD, Creutzfeldt–Jakob disease; CNS, central nervous system.

**Table 3:** Red flags: clinical features that might indicate a CJD mimic

**Feature**	**Indicates**
Fever	Infections, lymphoma
Seizures	Implies less likely to be CJD, infective and immune encephalitis, neoplasia, many other mimics
Hyponatraemia	VGKC encephalitis
Facial movement disorder	NMDA-R encephalitis, CNS Whipple’s disease
Modest progression	Implies less likely to be CJD, more likely to be a common neurodegenerative disorder
CSF pleocytosis	Infections, lymphoma, inflammatory, neoplastic
Contrast-enhancing lesions	Infections, lymphoma, inflammatory, neoplastic
MRI hyperintensities on T2-weighted imaging outside the striatum, thalamus and cortex	Vascular diseases, lymphoma, progressive multifocal leukoencephalopathy, encephalitis, extrapontine myelinolysis, others

CJD, Creutzfeldt–Jakob disease; CNS, central nervous system; CSF, cerebrospinal fluid; NMDA-R, *N*-methyl-D-aspartate; VGKC, voltage-gated potassium channel.

#### Toxic–metabolic syndromes

Most of these syndromes are picked up in the initial laboratory testing, which should include basic serum biochemistry, calcium, magnesium, glucose and thyroid function. It is important to emphasise the importance of *Wernicke’s encephalopathy*, an acute neurological disorder, precipitated by thiamine deficiency, characterised by the clinical triad of ophthalmoplegia, ataxia and confusion, which should be treated with urgent intravenous B vitamin replacement. Imaging features may partially overlap those of CJD. The encephalopathy associated with *hepatic failure* has mimicked CJD on a couple of occasions at our clinic, with the associated negative myoclonus (a sudden loss of muscle tone) in comatose patients mimicking the end stages of CJD. The detection of *hyperammoninaemia* either as part of rare genetic urea cycle disorders, drug therapy or liver failure can be crucial in the diagnosis of a toxic encephalopathy. In addition, a high signal in the lentiform nucleus on T1 MRI sequences due to manganese deposition strongly suggests hepatic encephalopathy.

#### Neoplastic and paraneoplastic conditions (other than limbic encephalitis)

The conditions hardest to distinguish are those with no or only subtle mass lesions on MRI. Typically, these include primary CNS lymphoma, carcinomatosis and intravascular lymphoma. In these cases, the imaging findings are usually the crucial diagnostic. *Primary CNS lymphoma* is usually associated with enhancing lesions in contact with CSF. Large-volume CSF sampling for cell analysis before a trial of corticosteroids may avoid brain biopsy. Serum tumour markers and whole-body imaging may also help. *Intravascular (B-cell) lymphoma* is a rare and notoriously difficult diagnosis. It leads to rapid cognitive decline, seizures, upper motor neurone signs and peripheral signs such as those of a neuropathy. Imaging findings overlap those of primary CNS vasculitis.

#### Vascular

We have seen cases of *large vessel stroke*, *dural arteriovenous fistula, posterior reversible encephalopathy syndrome* and *primary CNS vasculitis* as mimics of CJD. In each case, the MR imaging review was the main indicator of the diagnosis.

#### Mimics relevant to other types of prion disease

Genetic and iatrogenic forms of prion disease often do not progress as rapidly as CJD and therefore have distinct differentials. A common phenotype of several inherited prion disease mutations is a modestly progressive (over the years) cerebellar ataxia, often with a distal leg sensory disturbance, absent ankle jerks and extensor plantar responses. Iatrogenic CJD also commonly presents with ataxia and normal or near-normal cognitive function and is slightly slower in progression than typical sporadic CJD. Other forms of inherited prion disease have pure cognitive presentations, often with behavioural disturbance and are mistaken for the frontal variant of Alzheimer’s disease, Huntington’s disease (as there is an autosomal dominant family history) or frontotemporal dementia.

#### Conclusions and possible developments

RPD mimicking CJD is rare but requires urgent investigation and can be difficult to diagnose. This can be more rewarding than the investigation of insidiously progressing cognitive dysfunction, as there is a wide range of potentially treatable disorders. Specialist services for prion disease, including the NHS National Prion Clinic at the National Hospital for Neurology and Neurosurgery (University College London Hospitals NHS Trust) and the National CJD Research and Surveillance Unit at Edinburgh University, are here to help for assessment, to advise about local investigation and to provide access to molecular genetics and other diagnostics, follow-up and supportive care. We like to hear about cases as early as possible, certainly immediately after a scan showing changes compatible with CJD.

With such rare disorders, clinical research can most effectively be done when concentrated in national centres with long-term funding and provided alongside specialist care in partnership with local physicians. Research is delivering meaningful improvements, with specific blood and CSF prion disease diagnostics moving from the laboratory to clinical use in the last 5 years. It is hard to predict when we shall see any meaningful treatments, but this is certainly a major focus of activity at the moment. Progress in this area will not be possible without the continued referral and support of UK neurologists.

**Key points**

Creutzfeldt–Jakob disease (CJD) is a rapidly progressive and highly heterogeneous condition that readily mimics a wide range of potentially reversible or treatable disorders.
Highly sensitive and specific tests are available for CJD ­diagnosis, particularly diffusion-weighted MR imaging and cerebrospinal fluid (CSF) analysis using the real-time quaking-induced conversion assay; while the clinical syndrome can be mimicked, investigations should rapidly resolve any confusion.
Investigations are not as helpful for genetic forms of prion disease—clinicians should have a low threshold for ruling out this possibility by a gene test.
Be concerned about a diagnosis of CJD in the presence of unexplained fever, generalised seizures, hyponatraemia, a facial movement disorder, modest rates of progression (clinical duration >1 year), a CSF pleocytosis, contrast-enhancing MR lesions and white-matter lesions outside the striatum, thalamus and cortex.
